# Rhizosphere Diazotrophs in Acidic Agroecosystems: An Evidence-Chain and Microbiome-Compatibility Framework for Stabilizing Crop Growth Promotion

**DOI:** 10.3390/life16091460

**Published:** 2026-08-31

**Authors:** Qingye Yu, Yadi Yu, Lvshui Zhang, Wei Li, Hui Zeng, Kewen Gong, Feiyang Xiong, Qin Ying, Nansheng Wu, Ling Zhang

**Affiliations:** 1 College of Forestry, Jiangxi Agricultural University, Nanchang 330045, China; 13217930122@163.com (Q.Y.); yuyadi216@163.com (Y.Y.); zhanglvshui@jxau.edu.cn (L.Z.); liwei200428@163.com (W.L.); 19179039692@163.com (H.Z.); gongkewen1500@163.com (K.G.); 16679309317@163.com (F.X.); yingqinww@163.com (Q.Y.); 2 National Innovation Alliance of *Choerospondias axillaris*, Nanchang 330045, China

**Keywords:** acidic agroecosystems, rhizosphere diazotrophs, biological nitrogen fixation, root colonization, plant growth promotion, microbial consortia, synthetic microbial communities

## Abstract

Acidic soils are widespread in global agroforestry systems and severely constrain crop production through proton stress, aluminum/manganese phytotoxicity, phosphorus fixation, and rhizosphere microbiome reassembly. Rhizosphere diazotrophs can theoretically contribute to plant nitrogen (N) nutrition and stress adaptation. However, their N-fixation efficiency and growth-promoting effects in acidic soils are often highly unstable, limiting the predictability of field applications. This narrative mechanistic review synthesizes and critically interprets evidence on the physicochemical filters governing diazotroph survival in acidic soils, nitrogenase regulation, root-exudate-mediated recruitment, multi-guild microbial interactions, and the conditional design of synthetic microbial communities (SynComs) and inoculant formulations. We further discuss the potential complementary roles of phosphate-solubilizing bacteria (PSB) and arbuscular mycorrhizal fungi (AMF) and propose minimum reporting standards to improve field translatability. The synthesis indicates that effective diazotroph-mediated crop promotion depends on pH buffering, metal detoxification, carbon supply, phosphorus availability, host compatibility, and native microbiome receptivity. The proposed evidence-chain and microbiome-compatibility framework is a conceptual guide for evaluating these linked conditions rather than an empirically validated predictive model.

## 1. Introduction

In recent years, soil acidification has emerged as a critical constraint on sustainable crop production across diverse agricultural and agroforestry systems worldwide [1,2]. Acidic soils occur across many important agricultural and agroforestry regions, but their estimated extent depends on the pH threshold, land category, and mapping method used. Low soil pH impairs crop growth through multiple interconnected pathways: elevated H^+^ concentrations increase the solubility and phytotoxicity of Al^3+^ and Mn^2+^, inhibiting root elongation and lateral root formation [1]; phosphorus is immobilized through precipitation with aluminum and iron minerals [1]; and base cations such as calcium and magnesium are progressively leached, collectively depleting soil fertility [2]. Moreover, pH-induced shifts in rhizosphere chemistry alter root exudate profiles, oxygen diffusion rates, and carbon substrate availability, thereby restructuring the physicochemical gradients at the root–soil interface [3,4,5]. The cumulative effects of these abiotic stresses constrain crop productivity and alter the assembly, network architecture, and ecological functioning of rhizosphere microbial communities [3,4,5].

Against this backdrop of multifactorial stress, biological nitrogen fixation (BNF) is one of the microbial processes most closely linked to crop nutrient supply under low-input or nutrient-limited conditions. Diazotrophs can provide biologically fixed nitrogen and may also contribute to phosphorus mobilization, siderophore production, 1-aminocyclopropane-1-carboxylate (ACC) deaminase activity, phytohormone modulation, and induced systemic resistance. However, as in other soil-management systems, the presence of a beneficial trait does not necessarily mean that the trait will be expressed in the field; recent studies show that diazotroph diversity and activity vary with soil context, land management, nitrogen rate, and intercrop- or amendment-driven rhizosphere processes [6,7,8,9,10,11].

Recent studies have broadened this topic from single-strain descriptions to rhizosphere and microbiome management. Root exudates are increasingly considered chemical signals that recruit and assemble beneficial microbiota [3,4,12]. Synthetic microbial community (SynCom) studies emphasize functional complementarity, compatibility, and ecological stability [13,14,15], whereas microbiome-engineering frameworks connect inoculation, genome editing, and systems-level design [16]. As framework-level evidence, these advances are especially relevant for acidic soils because pH buffering, Al/Mn detoxification, P acquisition, and native-community resistance must be considered together.

In this narrative mechanistic review, we synthesize and critically interpret current knowledge on the potential contribution of rhizosphere diazotrophs to crop growth in acidic agroecosystems. We focus on the acid-soil filters that regulate diazotroph survival, colonization, and nitrogenase activity; the direct and indirect pathways through which diazotrophs may influence crop growth under acid stress; and the ways in which strain screening, SynCom design, and soil management can be integrated to improve field stability and ecological safety. We use the evidence-chain and microbiome-compatibility framework as a conceptual organizing device, not as a validated predictive model.

###  Narrative Review Scope and Inference Basis 

This article was prepared as a narrative and mechanistic review rather than as a formal systematic review. It does not claim a preregistered protocol, PRISMA-based exhaustive screening, a flow diagram, or a formal risk-of-bias assessment. During preparation, the authors consulted Web of Science Core Collection, Scopus, PubMed, Google Scholar, and Crossref for DOI metadata to identify literature relevant to acid-soil chemistry, diazotroph physiology, rhizosphere assembly, crop response, and inoculant design. Because the retrospective evidence-identification record was not designed as a reproducible database protocol, we do not report reconstructed database-specific yield counts or a screening flow. The literature published from 2020–2026 was emphasized, with 2025–2026 studies prioritized where available; older work was retained only for foundational evidence on acid tolerance, rhizobial symbiosis, biological nitrogen fixation, or nitrogenase regulation.

During narrative synthesis, evidence was interpreted according to its directness and contextual applicability. Studies were used for direct inference only when they reported diazotroph-related outcomes in a defined acidic soil setting; broader acidic soil PGPR, AMF, PSB, or microbial-community studies were retained only as indirect mechanistic support; and non-acid SynCom, AMF, root-exudate, or microbiome-engineering studies were treated as framework-level, hypothesis-generating evidence. Studies lacking a clear microbial or plant-response endpoint, adequate environmental context, or a direct connection to the claim under discussion were not used to support direct acidic soil diazotroph efficacy.

Recent reviews have focused on plant-growth-promoting SynCom design, AMF-mediated microbiome structuring, or broad plant microbiome engineering [13,14,15,16,17]. By contrast, the present review uses acidic soil chemical filters, diazotroph persistence and functional expression, evidence directness, native microbiome compatibility, and field-translatability criteria to connect these topics. This comparison defines the scope of the proposed framework without claiming that it replaces the evidence provided by those broader reviews.

## 2. Acidic Soil Rhizosphere Environment and Diazotroph Ecology

### 2.1. Physicochemical Characteristics of Acidic Soils

**Figure 1:**
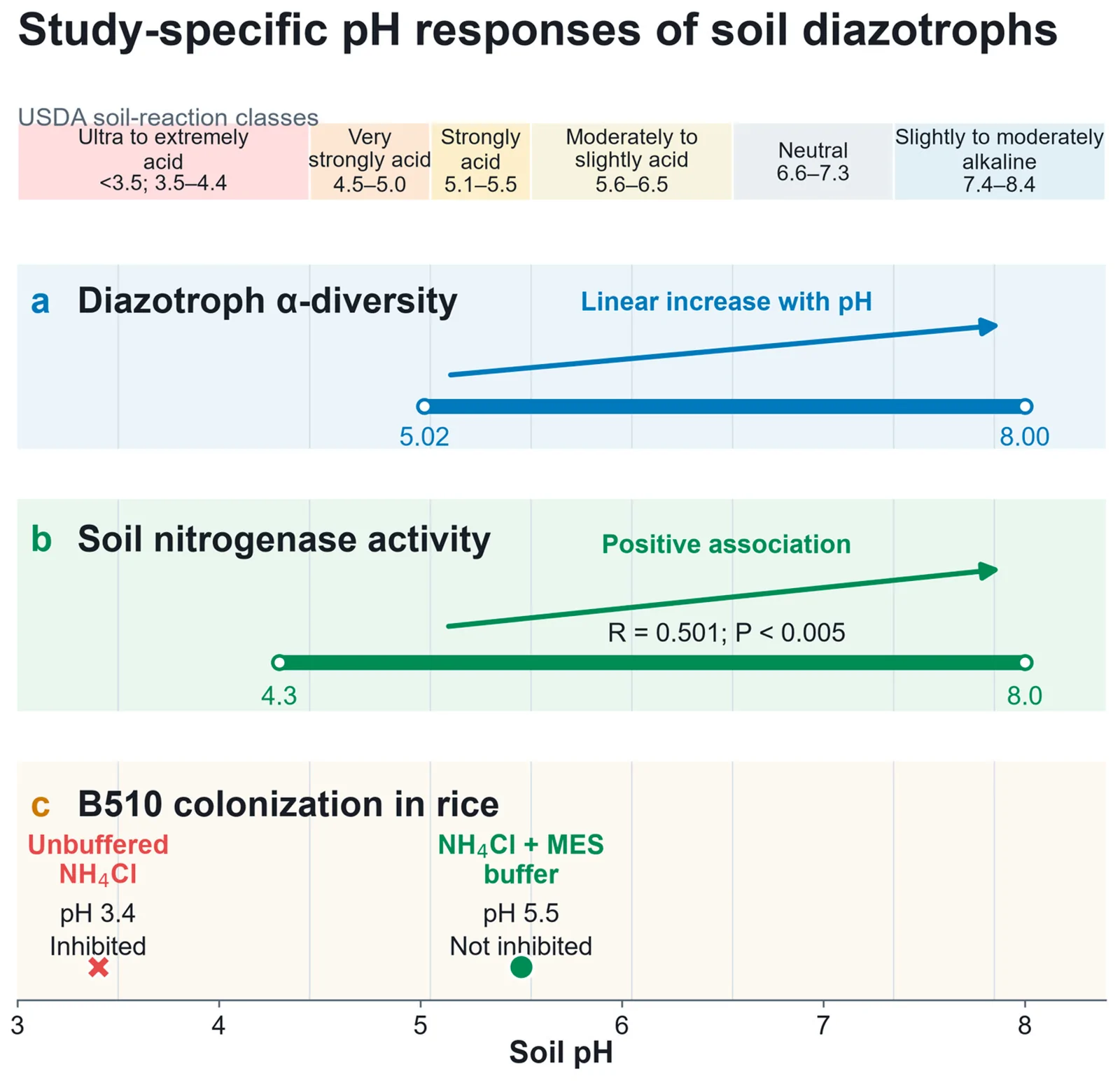
Study-specific pH responses of soil diazotrophs. Panel (**a**) shows diazotroph alpha diversity across Qinghai–Tibet alpine meadow soils [19]; panel (**b**) shows the association between soil pH and nitrogenase activity across Chinese upland soils [20]; and panel (**c**) shows pH-dependent colonization of rice by *Azospirillum* sp. B510 [21]. USDA soil-reaction classes are cited as descriptive terminology [22]. Arrows indicate reported directions only; no cross-study pooling, interpolation, or extrapolation was performed.

For synthesis, this review uses pH < 5.5 as a broad acidic soil category and pH < 4.5 as a severe-acidity subset [1,18]. The soil-reaction classes shown in Figure 1 provide descriptive context rather than universal biological thresholds. Figure 1 presents three study-specific pH responses selected to illustrate community diversity, nitrogenase activity, and root colonization under contrasting systems; the panels do not form a unified response curve and were not quantitatively pooled, interpolated, or extrapolated.

Acidic soils are characterized by low pH, altered nutrient availability, reduced cation exchange capacity, and increased solubility of aluminum and manganese. These changes decrease the availability of effective phosphorus and other nutrients, increase metal stress to plant roots, and restrict root extension, soil aeration, and water–nutrient acquisition [1,2]. Acidification constrains plant nutrition through both direct and microbial pathways. On the abiotic side, P becomes immobilized by Al/Fe minerals and root growth is restricted by metal toxicity. On the biotic side, nutrient-cycling microorganisms must maintain activity in microsites with fluctuating pH, oxygen, and carbon supply [1,23,24]. Recent phosphate-solubilizing bacteria (PSB) literature emphasizes acid-mediated inorganic P solubilization, organic P mineralization, and root–microbe interactions as key mechanisms for improving P availability, which is particularly relevant because N fixation and plant N assimilation are constrained when P is limiting [23].

### 2.2. Formation of the Rhizosphere Microenvironment

Root activity is a major driver of rhizosphere microenvironment formation. Through respiration and the secretion of organic acids, sugars, amino acids, phenolics, flavonoids, and other secondary metabolites, roots change carbon supply, acid-base status, and chemical signaling around the root surface. These compounds can support diazotroph recruitment, chemotaxis, biofilm formation, and functional expression, but their effects in acidic soils depend on pH buffering, Al/Mn toxicity, P availability, and host-specific exudation patterns [1,3,4,5,9,12,25].

Soil physicochemical properties, such as texture, organic matter content, and matrix buffering capacity, determine the scope and intensity of root exudate influence. In acidic soils, lower buffering capacity makes root-secreted organic acids more likely to shift local pH, thereby altering metal ion solubility, nutrient availability, enzyme activity, and bacterial-community structure [1,24]. The micropores created by root growth and the water dynamics around roots alter oxygen diffusion and moisture gradients, further affecting microbial metabolic pathways and community assembly.

Competition and cooperation among rhizosphere microorganisms determine whether recruited diazotrophs persist long enough to affect plant performance. In acidic soils, native-community resistance, protozoan grazing, bacteriophage pressure, antagonism among inoculant partners, and priority effects can prevent introduced strains from occupying the root niche even when they possess favorable in vitro traits; conversely, compatible resident networks can support nutrient cycling and stress buffering [11,13,16,26].

### 2.3. Classification and Ecological Functions of Diazotrophs

Diazotrophs are microorganisms capable of reducing atmospheric nitrogen into forms usable by plants, including nitrogen-fixing cyanobacteria, actinomycetes such as *Frankia*, certain archaea, and diverse obligate or facultative nitrogen-fixing bacterial groups across multiple phyla, including major Proteobacterial lineages [27,28]. Global-scale soil surveys show that diazotroph diversity and distribution are structured by broad environmental and biogeographic filters, reinforcing the need to interpret acidic soil diazotrophs within both local rhizosphere niches and larger microbial-distribution patterns [27]. Based on ecological niches and lifestyles, diazotrophs include symbiotic strains associated with plants, free-living nitrogen-fixing microorganisms in rhizosphere environments, and endophytic diazotrophs that colonize internal plant tissues [27,29,30,31].

Diazotrophs from different taxonomic groups differ in nitrogenase systems, electron-donor acquisition pathways, and sensitivity to oxygen and pH [1,28,32]. Nitrogenase catalysis requires ATP hydrolysis and low-potential electron transfer from the nitrogenase reductase to the catalytic component; the carbon, redox, oxygen, nutrient, and stress physiology that supplies these requirements differs among organisms. Alternative Vnf and Anf nitrogenases occur in some diazotrophs, but the evidence reviewed here does not establish their ecological relevance in acidic agricultural rhizospheres; they are therefore acknowledged as a scope limitation rather than used to infer field performance. These physiological differences affect ecological distribution, rhizosphere competitiveness, and functional performance in acidic soil environments.

Plant-associated diazotrophs and related PGPRs may influence root development and rhizosphere carbon flow through phytohormone-related processes, phosphorus mobilization, siderophore production, ACC–deaminase activity, and other context-dependent functions [23,33,34,35]. Detailed discussion of root development and stress responses is reserved for Section 4.2 and Section 4.3 to avoid repeating the same ecological functions across sections.

Certain diazotrophs may contribute to plant stress resistance, whereas broader PGPR and metal-stress studies indicate related mechanisms such as induced systemic resistance, antioxidant activity, and metal-chelating compounds [33,36,37,38,39]. These mechanisms may help alleviate common stresses in acidic soils, including aluminum toxicity, heavy metal stress, and oxidative damage. Reciprocal filtering between plant root exudates and diazotrophs is a key determinant of community composition and functional expression [3,4,9,12,25]. Carbon-source availability, pH gradients, and stress-induced exudate shifts jointly shape whether diazotrophs are recruited, retained, or excluded in acidic rhizospheres [3,4,25]. These ecological strategies and evidence limitations are summarized in Table 1.

### 2.4. Effects of Acid Stress on Rhizosphere Diazotrophs

Acid stress affects rhizosphere microorganisms through both chemical and biological pathways. Low pH changes nutrient availability, metal toxicity, and root exudation, thereby inhibiting sensitive microbial populations, selecting for acid- and metal-tolerant taxa, and altering the balance among diazotrophs, phosphate-solubilizing bacteria, AMF, and pathogens [1,2,3,4,5,24,27]. Recent evidence further indicates that acid stress reorganizes rhizosphere microorganisms through selective enrichment rather than uniform suppression. Management practices that alter organic inputs, pH buffering, nutrient supply, and PGPR-mediated nitrogen cycling can therefore change whether diazotroph functions are retained, lost, or replaced by other microbial guilds [7,11].

Evidence from non-acidic or desert systems should be used cautiously in this review. Such studies are useful for illustrating environmental filtering, SynCom assembly, or microbiome-engineering concepts, but they cannot by themselves support claims about acidic soil diazotroph function unless pH, Al/Mn toxicity, nutrient status, host background, and microbial endpoints are reported [1,13,14,15,16,17,18,42]. A more rigorous interpretation is that low pH filters the community through metal toxicity, carbon allocation, nutrient limitation, and biotic interaction networks. This explains why some acid-tolerant taxa become enriched while overall functional reliability can still decline when the community loses complementary diazotroph diversity [1,5,11].

## 3. Colonization and Nitrogen Fixation Mechanisms of Diazotrophs in Acidic Soils

### 3.1. Rhizosphere Colonization Process and Signal Recognition

Rhizosphere colonization is the first step by which diazotrophs can affect crop growth [24,26,41,42]. This process includes chemotactic movement toward root-derived compounds, attachment to the root surface, biofilm formation, and molecular communication with the host [3,4,24,25,41,42]. Acidic pH and soluble metals may alter signal diffusion, membrane stability, and competition on the root surface; the direct consequences for diazotroph colonization require verification in target acidic soil systems [1,24,30,39]. Low-molecular-weight organic acids, sugars, amino acids, phenolics, and flavonoids in root exudates can shift under nutrient deficiency and acid–metal stress [1,3,4,5,24]. These shifts can recruit beneficial bacteria and fungi, but the direction of recruitment is host-, soil-, and community-dependent. Non-acid root-exudate studies are used here to formulate mechanisms rather than to prove acidic soil diazotroph efficacy [1,3,4,5,12,25].

Signal recognition between roots and diazotrophs involves plant-derived exudates, microbial quorum signals, and host immune modulation. Acidic pH and soluble metals can alter the stability and diffusion of these signals, while AMF hyphae and hyphosphere bacteria may expand the spatial scale of chemical communication. Thus, colonization should be interpreted as a multi-partner process rather than as a binary attachment event between a single strain and a root surface [3,17]. The presence of other rhizosphere microorganisms can inhibit diazotroph colonization through niche overlap and antagonism, but it can also promote colonization through cross-feeding, co-aggregation, and stress buffering [11,13,16,26,42]. This duality explains why single-strain inoculants often perform well in vitro but fail in non-sterile acidic soils, and why compatibility testing with the resident microbiome is now considered essential for inoculant development [11,13,16].

### 3.2. Nitrogenase System and Nitrogen Fixation Pathways

Nitrogenase is the key enzyme system that reduces atmospheric N_2_ to plant-available ammonia [28,32]. In acidic soils, however, nitrogenase activity is controlled not only by enzyme capacity but also by carbon and electron supply, oxygen protection, micronutrient availability, cellular pH homeostasis, P status, and the host–rhizosphere microenvironment [1,7,10,11,24,28,32]. Field-oriented evidence further indicates that reduced diazotroph diversity can decrease free-living nitrogen fixation rates, although the magnitude of this effect depends on land-management context [11].

**Figure 2:**
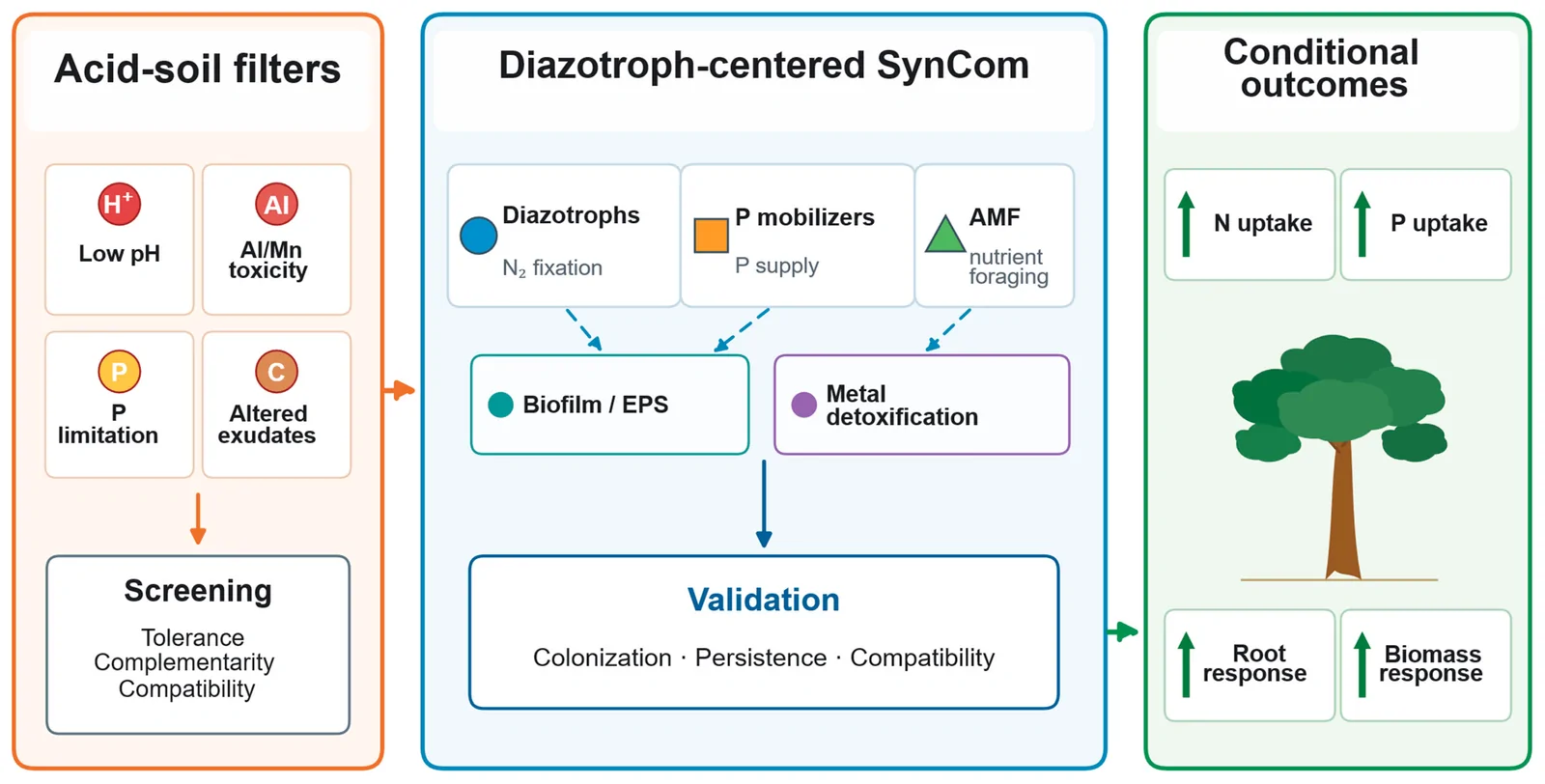
Evidence-informed design of diazotroph-centered SynComs for acidic soils. Acid-soil filters guide candidate selection, while plant outcomes remain conditional on colonization, persistence, and native microbiome compatibility. Biofilm/EPS and metal-detoxification traits provide complementary support. Dashed arrows indicate proposed or conditional links rather than validated quantitative interactions [1,3,4,5,7,12,13,14,15,16,17,23,38,39,41]. The green upward arrows indicate an increase.

Consequently, nitrogenase activity is necessary but not sufficient for plant growth promotion. Without persistent colonization, adequate plant carbon allocation, P availability, and protection from metal-induced oxidative stress, high acetylene-reduction activity in culture may not produce measurable field benefits [1,7,11,28,32]. This distinction is important for interpreting Figure 2 and for avoiding over-generalization from a small number of model strains. A major limitation in the current literature is the lack of comparable measurement systems. Acetylene-reduction assay (ARA) values, nifH abundance, ^15^N incorporation, colonization density, and plant biomass response are often reported under different pH, carbon, oxygen, host, Al/Mn, and P conditions [1,2,7,8,10,11,27,28,32]. These indicators should therefore be treated as separate links in an evidence chain rather than pooled into a single efficacy ranking.

### 3.3. Adaptive Regulatory Mechanisms in Acidic Soil Environment

In acidic environments, diazotrophs face multiple stresses such as low pH, aluminum and manganese toxicity, and reduced nutrient availability, thus requiring multi-level adaptive regulation to maintain survival and nitrogen fixation function [1,2,7,30]. At the community level, root exudates, amendments, AMF, and PSB can buffer or intensify these stresses depending on local compatibility.

At the cellular level, acid-adapted diazotrophs and related acid- or metal-tolerant PGPRs may maintain function through proton extrusion, membrane remodeling, antioxidant protection, extracellular polymeric substances, siderophore-mediated metal chelation, and biofilm-mediated microsite stabilization [1,30,33,36,38,39,41]. At the plant level, recent work on aluminum toxicity shows that PGPR can interact with auxin transport and stress-hormone pathways, indicating that acid tolerance and growth promotion are coupled through plant hormonal networks rather than being independent bacterial traits [38]. These mechanisms are promising, but the evidence remains concentrated in a limited set of host–strain systems.

At the community level, AMF and other microbial guilds can alter nutrient acquisition, root signaling, secondary metabolism, and rhizosphere architecture. The AMF-based SynCom literature indicates that fungal hyphae may structure bacterial partners and nutrient exchange, but it does not by itself demonstrate direct AMF–diazotroph interaction [15,17,43]. For acidic soil diazotrophs, these findings are treated as design hypotheses that require direct validation under reported pH, Al/Mn, P, and host conditions.

### 3.4. Synergistic Interactions with Other Rhizosphere Microorganisms

Diazotroph function in acidic soils should be considered within a microbial network rather than as an isolated bacterial trait [1,7,11,13,16,26,42]. PSB can increase P availability and thereby support N assimilation [23,35,44]; AMF can expand nutrient-foraging zones and alter root signaling [15,17,43,45]; organic matter-decomposing bacteria can modify carbon flow [46]; and antagonistic microorganisms can reduce pathogen pressure [3,4,33,37,42]. These interactions are beneficial only when partner compatibility and native-community acceptance are maintained.

In acidic rhizosphere environments, diazotroph-containing microbial assemblages may benefit from cooperation with phosphate-solubilizing bacteria, organic matter-decomposing bacteria, and antagonistic microorganisms: phosphate-solubilizing bacteria release available phosphorus that can support nitrogen assimilation; organic matter decomposers influence carbon flow and nutrient release; and antagonistic microorganisms may reduce pathogen pressure, thereby potentially creating a more favorable niche for nitrogen fixation activities [7,23,35,37,40,42]. Different functional groups can also reshape root-exudate profiles, root development, and symbiotic regulation, thereby affecting host adaptation to low-pH stress [1,3,4,6,9,15,17,25,32,40].

For practical application, single-strain inoculation should be regarded as only one option within a broader microbiome-management strategy. Site-adapted consortia may be more reliable than untested strain mixtures when they are designed around functional complementarity, low antagonism, host compatibility, and tolerance to local pH, Al/Mn, moisture, and nutrient conditions [1,13,14,15,16,42]. However, increasing community complexity can also create new risks, including priority effects, unpredictable succession, horizontal gene transfer, and inconsistent dominance of target strains. Taken together, colonization and nitrogenase activity should be treated as necessary conditions rather than final success criteria [1,7,11,24,28,32,41]. A robust acidic soil inoculant should therefore be evaluated through a chain of evidence: survival under target pH and metal concentrations, stable root colonization, functional expression of nifH or nitrogenase activity, plant N/P uptake, yield or root response, and persistence within the native microbiome.

## 4. Diazotroph-Mediated Crop Growth Promotion

### 4.1. Contribution of Biological Nitrogen Fixation to Crop Nitrogen Nutrition

Diazotrophs directly supplement the nitrogen source for crops in acidic soils by converting atmospheric nitrogen into forms absorbable and utilizable by plants [7,28,32]. This contribution is particularly significant under conditions of insufficient soil nitrogen supply, but its agronomic value depends on root colonization, nitrogenase protection, available P, plant demand for N, background N input, and diazotroph community adjustment [7,8,10,11].

Root exudates regulate both community assembly and functional expression of diazotrophs by supplying carbon, shaping redox microsites, and acting as chemical signals [3,4,5,9,12,24,25]. Intercrop and model–rhizosphere studies indicate that exudates can co-drive nitrogen fixation and productivity through key rhizosphere bacteria, yet recent plant-exudate reviews caution that recruitment is not universally beneficial: exudates can also favor competitors, pathogens, or poorly compatible microbes [3,4,9,12,25]. In acidic soil diazotroph management, this literature is therefore used to define testable recruitment mechanisms rather than direct efficacy claims.

Recent findings indicate that the contribution of diazotrophs to plant nitrogen nutrition should be evaluated at the community level. Reduced diazotroph diversity can depress nitrogen fixation rates, but the response depends on land management, implying that soil amendment, crop rotation, organic inputs, and inoculation history can determine whether diazotroph functions are redundant or vulnerable [11]. Therefore, biological nitrogen fixation should be discussed together with P availability, root carbon allocation, and microbial network stability [3,4,7,11,23,24,28,32,45]. In acidic soils, a strain with high N-fixing potential may remain agronomically ineffective if P limitation, Al/Mn toxicity, or native-community exclusion prevents expression of that potential at the root–soil interface [1,2,7,11,24].

### 4.2. Phytohormone Regulation and Root Development Promotion

Evidence from diazotrophs and broader PGPR systems indicates that microbial inoculants can regulate root development by modifying phytohormone balance and stress signaling [33,36,37,38]. PGPR-mediated auxin, cytokinin, abscisic acid, jasmonate, and ethylene-related pathways can alter lateral root formation, root-hair density, antioxidant capacity, and Al-stress response, but these outcomes depend on pH, host genotype, nutrient status, and native-community context [36,37,38]. Nevertheless, hormone-related promotion should not be treated as a universal outcome of inoculation. The same auxin-producing or ACC–deaminase-positive strain may produce different phenotypes under different pH, host genotype, nutrient, and microbiome contexts [1,11,16,36,37,38,42]. This explains why screening must include plant phenotypes under realistic acidic soil conditions rather than relying solely on in vitro biochemical traits [1,38,47].

### 4.3. Stress Resistance Induction and Crop Adaptability Enhancement

Diazotrophs may contribute to crop adaptation in acidic soils by improving nutrient acquisition and stress tolerance, while broader PGPR evidence indicates related mechanisms involving antioxidant defenses, ethylene regulation, metal chelation, EPS, siderophores, ACC deaminase, and plant stress–hormone cross-talk [1,33,35,36,37,38,39]. Direct acidic soil diazotroph evidence should be separated from broader PGPR stress-tolerance evidence. Recent EPS-focused research further supports this interface perspective: extracellular polymeric substances produced by PGPR can condition soil and contribute to positive plant–soil feedback, suggesting that inoculant performance may depend on whether microbial products persist and alter local soil structure, water retention, and nutrient exchange [41].

Taken together, stress-resistance induction should be framed as plant–microbe–soil feedback rather than as an isolated bacterial trait. Current evidence is strongest for controlled or semi-controlled systems, whereas long-term field validation under combined acidic soil stresses remains limited. This limitation should temper claims about universal inoculant efficacy [1,7,11,13,14,16,42]. For acidic soils, this pathway should be validated by linking microbial trait assays to plant stress markers, nutrient uptake, root traits, and yield response under reported pH-Al/Mn-P conditions.

### 4.4. Strain Screening, Inoculation Effects, and Application Models

Field application of diazotrophs should begin with strains or consortia that retain function under target acidic soil chemistry [1,2,7]. Candidate inoculants need to be tested for acid and metal tolerance, nitrogenase stability, root colonization, host compatibility, interaction with native communities, formulation survival, and performance under realistic pH-Al/Mn-P constraints; recent strain-screening studies provide useful examples but still need stronger field-context reporting [47,48]. A practical screening pipeline should therefore include: (i) laboratory stress assays under target pH, Al/Mn, and low-P conditions; (ii) plant-based assays that quantify colonization, N fixation, root traits, nutrient uptake, and stress markers; (iii) compatibility assays among candidate strains and with representative native microbiota; (iv) formulation tests for shelf life, carrier protection, and release dynamics; and (v) multi-site field trials with ecological monitoring.

The native microbiome background is not merely a source of variability but a determinant of inoculation success [7,11,13,16,26,42]. Priority effects, niche pre-emption, grazing, antagonism, and horizontal gene transfer can all reshape the fate of introduced strains [13,14,16,42]. Therefore, laboratory performance must be followed by compatibility tests against representative native microbiomes from target acidic soils. Overall, application should be organized as an iterative feedback loop: functional confirmation, compatibility testing, formulation optimization, site-adapted field validation, risk assessment, and post-application monitoring. Lime, organic amendments, and crop rotation may improve inoculant establishment, but they should be tested as part of the same system because they also alter microbial networks and nutrient availability [1,2,7,11,40]. The resulting inference-basis evidence map is presented in Table 2.

## 5. SynComs for Acidic Soil Agriculture: Conditional Solutions Rather than Universal Upgrades

For acidic soil agriculture, SynCom design should be based on clearly defined target functions, including N fixation, P mobilization, Al/Mn detoxification, hormone-mediated root remodeling, pathogen suppression, and persistence in the native microbiome [1,7,13,14,16,23,35,37,38,39,42]. Recent SynCom and functional microbiome literature emphasizes functional complementarity, compatibility testing, ratio optimization, ecological monitoring, and model-guided interaction analysis, but these principles require validation under target-soil pH, Al/Mn, P, host genotype, and colonization conditions [1,5,12,13,14,15,16,42].

AMF-based SynComs may be particularly relevant because hyphal networks can extend nutrient foraging, structure hyphosphere bacterial communities, and provide spatial continuity for microbial interactions, although these functions still require validation in acidic diazotroph-containing systems [15,17,43,45]. However, SynComs should not be presented as inherently superior to single strains. Failure modes include unstable assembly, dominant-strain overgrowth, host- and soil-dependent performance, formulation instability, regulatory uncertainty, and native-community resistance [13,14,16,42]. Therefore, SynComs should be evaluated as conditional designs whose success depends on the acid-stress niche fit and microbiome compatibility rather than on the number of assembled members.

Accordingly, Figure 2 is organized as a qualitative, three-stage design framework rather than a validated interaction network. Acid–soil filters (low pH, Al/Mn toxicity, P limitation, and altered root exudates) guide screening for tolerance, functional complementarity, and compatibility. Diazotrophs are then combined with P mobilizers, AMF, and supporting biofilm/EPS or metal-detoxification traits, while N and P uptake, root response, and biomass response are treated as conditional on demonstrating colonization, persistence, and native microbiome compatibility. The dashed arrows indicate proposed or conditional links and do not encode measured interaction strengths or a universal SynCom composition [1,3,4,5,7,12,13,14,15,16,17,23,38,39,41].

## 6. Synthesis and Application Perspectives

### 6.1. Acid-Soil Factors Regulating Diazotroph Performance

Acidic soils regulate diazotroph performance through interacting filters rather than through pH alone [1,2,5,7,11,18,24]. Low pH increases Al^3+^ and Mn^2+^ toxicity, reduces P and base-cation availability, alters oxygen and moisture microsites, and changes root exudate chemistry [1,2,5,24]. Iron availability and speciation are likewise pH-dependent and should be interpreted as a contextual plant- and microbe-nutrition variable rather than as a uniformly limiting factor. Together, these filters determine whether diazotrophs survive, colonize roots, protect nitrogenase, and compete with native microorganisms. The most defensible conclusion is conditional: acid-tolerant traits are necessary, but their realized function depends on host genotype, soil buffering capacity, land management, micronutrient context, and the resident microbiome [1,3,4,7,11,24].

### 6.2. Diazotroph-Mediated Crop Growth Promotion Under Acid Stress

Crop growth promotion under acid stress emerges from an integrated pathway network. Biological N fixation contributes to N supply, but growth responses may also depend on hormone-mediated root remodeling, phosphorus mobilization, siderophore-mediated metal chelation, EPS/biofilm protection, ACC–deaminase-mediated ethylene regulation, and induced defense [1,7,11,23,33,35,36,37,38,39,41]. Because these pathways can compensate for or constrain one another, isolated measurements such as ARA, IAA production, or nifH abundance should not be interpreted as direct predictors of yield unless linked to plant phenotype under target soil conditions [7,11,23,33,35,36,37,38,39,41].

### 6.3. Strain Screening, SynCom Design, and Soil Management for Field Stability

**Figure 3:**
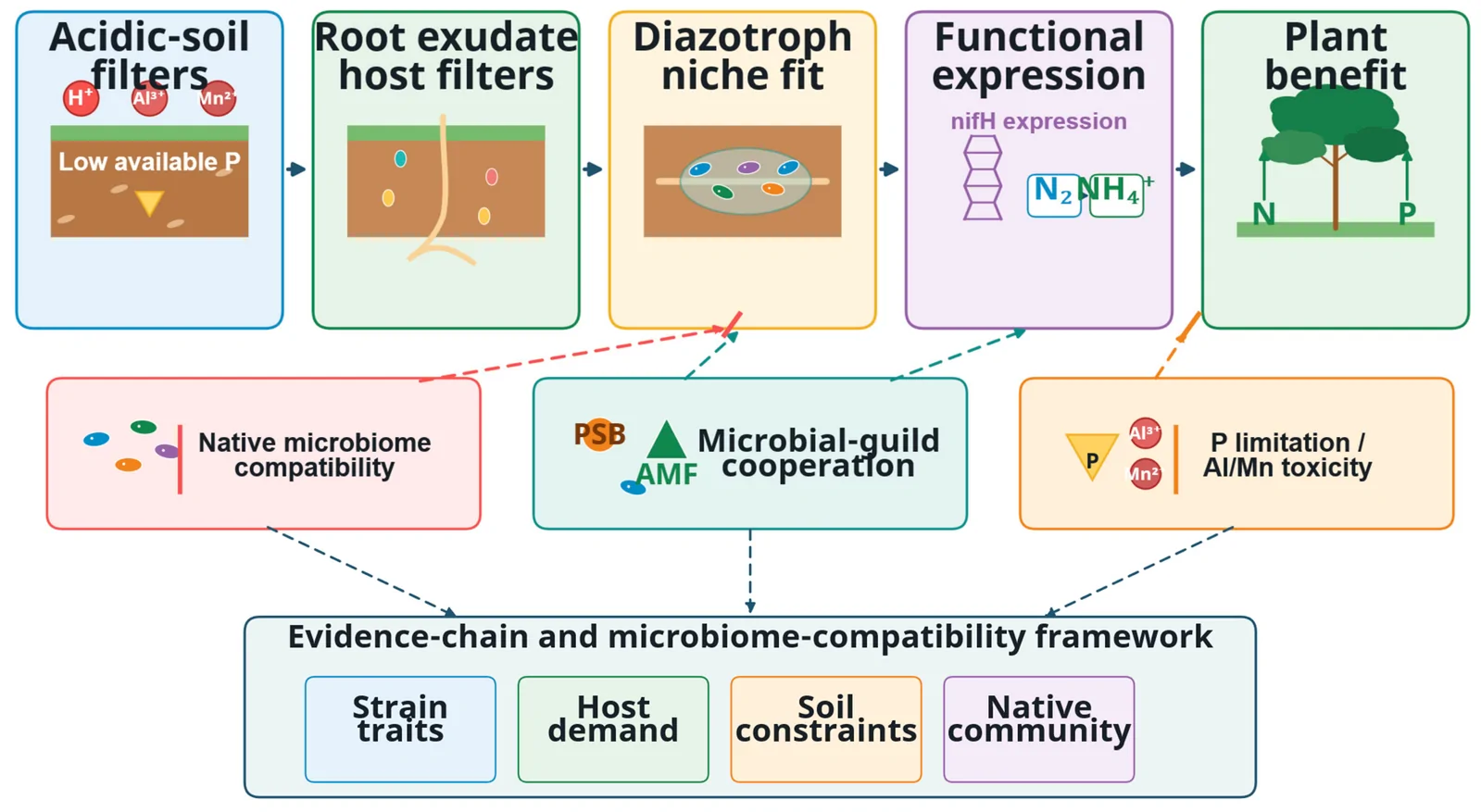
Proposed conceptual evidence-chain framework for acidic soil diazotroph management. Solid arrows denote conceptual pathways; dashed arrows denote feedback or framework-level inference. The figure does not represent an empirically validated model or quantified interaction strengths.

Field stability requires a shift from trait-first screening to system-level design. Strains should be selected for target-soil stress tolerance and then tested for host compatibility, community compatibility, formulation stability, and persistence under amendment regimes [1,2,7,13,14,16,42,47,48]. SynComs may improve functional redundancy and resilience when designed through complementarity and compatibility, but their ecological dynamics must be monitored because community succession can undermine intended functions [13,14,15,16,42]. Therefore, lime or organic amendment, inoculant formulation, SynCom composition, AMF/PSB cooperation, and ecological-risk assessment should be treated as an integrated management package [1,2,7,13,14,15,16,17,23,40,43,45]. Together, these three lines of evidence indicate that acidic soil diazotroph management is not a search for a universally superior strain. It is a context-dependent strategy for matching microbial traits, host demand, soil constraints, and native microbiome structure. This evidence-chain logic converts a mechanism summary into a testable microbiome-compatibility framework for acidic agroecosystems (Figure 3).

### 6.4. Theoretical and Practical Implications

The theoretical implication of this synthesis is that rhizosphere nitrogen fixation in acidic soils should be modeled as a coupled plant–soil–microbiome process [1,3,4,5,7,11,24]. The relevant explanatory variables include soil pH, Al/Mn toxicity, available P, root exudate composition, host genotype, diazotroph diversity, and multi-guild microbial interactions [1,2,3,4,5,7,11,18,24]. The practical implication is that inoculant development should avoid a one-size-fits-all strategy and instead match strain or SynCom traits to local soil constraints and crop demand. Product development should therefore prioritize acid-tolerant and metal-tolerant strains with verified colonization capacity, but it should also test compatibility with PSB, AMF, organic amendments, lime, and the resident microbiome. Composite inoculants may improve stability only when interaction compatibility and field persistence are verified. Otherwise, additional strains may increase unpredictability rather than reliability. Overall, this review calls for a tighter feedback mechanism between mechanism research and field application (Table 3), including standardized reporting of soil chemistry, inoculum traits, colonization data, N-fixation indicators, plant phenotypes, and ecological monitoring endpoints [1,2,7,11].

## 7. Conclusions

Rhizosphere diazotrophs may contribute to crop performance in acidic agroecosystems, but their agronomic relevance depends on linked evidence for survival, root colonization, nitrogenase expression, plant nutrient uptake, yield response, and compatibility with the resident microbiome. The evidence-chain and microbiome-compatibility framework is therefore proposed as a conceptual guide for testing these conditions, not as proof of universal inoculant efficacy.

## 8. Research Limitations and Future Prospects

Current evidence on diazotroph–crop interactions in acidic soils remains uneven, with direct field data limited; broader PGPR, AMF, SynCom, or microbiome-engineering studies should only be used as mechanistic support when their inference boundaries are explicit [1,2,3,4,5,15,16,17,23,42]. Future research should move beyond pH-only explanations toward joint experimental testing and reporting of pH, exchangeable Al/Mn, available P, root-exudate context, inoculant traits (colonization, survival, nitrogenase expression), plant N/P uptake, yield/root traits, native microbiome compatibility, and realized N fixation, linking multi-omics, isotope tracing, and culture-based validation as a coherent evidence chain [1,2,3,4,5,15,16,17,23,42] (Table 1, Figure 1).

To develop field-ready strategies, SynCom design should shift from functional stacking to acid-stress niche design, assembling consortia around complementary acid tolerance, P mobilization, metal detoxification, colonization niches, and native community compatibility, with field-scale validation connecting microbial persistence, N fixation, plant response, and ecological safety. We further propose minimum reporting standards (Table 4) to ensure comparability across soils, hosts, and inoculant designs, providing a testable framework for designing acid-stress-adapted inoculants and comparing future field studies across acidic agroecosystems.

## Figures and Tables

**Table 1:** Comparison of ecological strategies, acid-soil mechanisms, and evidence limitations among major rhizosphere diazotroph types.

Diazotroph Type	Representative Taxa	Acid-Soil Functional Strategy	Inference Strength and Limitations
Symbiotic rhizobia	*Bradyrhizobium*, *Rhizobium*, *Sinorhizobium*	Host-specific nodulation, leghemoglobin-mediated O_2_ buffering, extracellular polymeric substances (EPS) production, acid-adapted nod-gene regulation, and Al^3+^-tolerant symbioses such as B. pelecinus rhizobia [30,31,32,40]	Inference basis A where acidic soil nodulation or acid/Al tolerance is directly reported; otherwise, B for related rhizobial mechanisms.
Associative/free-living diazotrophs	*Azospirillum*, *Azotobacter*, *Burkholderia*	Root-surface attachment, biofilm formation, cytoplasmic pH homeostasis, membrane remodeling, EPS/biofilm buffering, antioxidant protection, siderophores, ACC deaminase, and P solubilization [23,33,35,36,39,41]	Inference basis A/B; direct acidic soil diazotroph reports should be separated from general PGPR evidence.
Endophytic diazotrophs	*Gluconacetobacter*, *Herbaspirillum*, *Azoarcus*	Internal root or shoot colonization, partial host-tissue buffering, strain-specific acid-adapted enzymes, IAA/gibberellin/ABA modulation, and induced stress resistance [29,38]	Inference basis B/C because many reports lack comparable pH, Al/Mn, inoculum density, and host-response metadata; field persistence remains insufficiently verified.

Inference bases A, B, and C were developed iteratively by the authors during narrative synthesis as a qualitative inference-control guide; they were not prespecified, independently duplicated, or used as a formal risk-of-bias tool. Basis A denotes direct acidic soil diazotroph evidence. Basis B denotes acidic soil PGPR, AMF, phosphate-solubilizing bacteria, or microbial-community evidence that provides indirect mechanistic support. Basis C denotes non-acid SynCom, AMF, root-exudate, or microbiome-engineering evidence used only for framework-level extrapolation. Ambiguous studies were conservatively assigned to B or C rather than used alone to infer acidic soil diazotroph efficacy. Abbreviations used in Table 1: EPS, extracellular polymeric substances; ACC, 1-aminocyclopropane-1-carboxylate; IAA, indole-3-acetic acid.

**Table 2:** Evidence map for acidic soil diazotroph and supporting microbiome studies.

Strain/Group	Crop/Host	Soil pH	Al/Mn Reported	Nitrogen-Fixation Metric	Colonization Metric	Plant Response	Inference Basis
Soil diazotroph community under organic/inorganic amendments [7]	Crop in acidic soil	Acidic soil reported	Soil acidity context reported; Al/Mn/P metadata should be explicit	Contribution to crop N utilization	Community-level diazotroph response	N utilization response to amendments	A
Acid/Al-tolerant rhizobia, including *Biserrula pelecinus* root-nodule bacteria [30,31]	Legume host	Low-pH tolerance reported	Al tolerance discussed; Mn often incomplete	Symbiotic biological nitrogen fixation (BNF)/nodulation	Nodulation and host compatibility	Soil-fertility restoration potential	A
Endophytic diazotrophs associated with water yam [29]	Dioscorea alata	Not consistently reported as acidic	Not reported	Endophytic occurrence and BNF-related indicators	Internal association/endophytic occurrence	Association with water-yam growth; acid-soil inference limited	C
PGPR/PSB/rhizobium nutrient-mobilization systems [23,35,44]	Chickpea and diverse crops	Variable or study-specific	Often incomplete	BNF or N-uptake support where measured	Inoculation/PGPR establishment	Improved N/P acquisition and growth	B
AMF-PSB-rhizobia cooperation [15,17,23,40,45]	Peanut, rice, and model systems	Variable	Often incomplete	Indirect support via nutrient acquisition	Hyphal scaffolding/crosstalk	Nodulation, P uptake, or soil-health response	B/C
SynCom and microbiome-engineering frameworks [13,14,16,42]	Diverse/model systems	Usually non-acid or not target-specific	Usually not acid–metal chemistry	Functional potential, not direct BNF proof	Assembly and compatibility metrics	Design principles; not direct efficacy proof	C

**Table 3:** Iterative pathway and decision points from acid-tolerant strain screening to field application in acidic soils [1,2,7,11,13,14,15,16,17,42,47,48].

Stage	Key Actions	Challenge and Mitigation
1. Strain and trait screening	Test target pH, Al/Mn tolerance, ARA or ^15^N_2_ fixation, IAA, siderophore, ACC deaminase, P solubilization, and EPS/biofilm traits.	Challenge: In vitro traits may not predict plant response. Mitigation: Advance only strains that retain function under target acidic soil chemistry.
2. Plant and microbiome validation	Quantify colonization, root phenotype, N/P uptake, stress markers, and interaction with representative native microbiota.	Challenge: Native competition and priority effects can exclude introduced strains. Mitigation: Reject incompatible strains or redesign consortia around local microbiome constraints.
3. SynCom and formulation design	Combine diazotrophs with PSB, AMF, or stress-buffering guilds based on complementarity, low antagonism, and host compatibility.	Challenge: Higher diversity can create unstable succession or dominant-strain overgrowth. Mitigation: Use compatibility assays, ratio optimization, carrier protection, and shelf-life tests.
4. Site-adapted field application	Run multi-site trials with lime/organic amendments, seed coating or soil drench, and crop-stage-specific timing.	Challenge: Controlled-environment efficacy may fail under field heterogeneity. Mitigation: Measure plant yield, N-use efficiency, colonization persistence, and soil pH/P/Al/Mn changes.
5. Ecological monitoring	Track native microbial networks, target-strain persistence, non-target effects, and functional stability after application.	Challenge: Ecological risk and long-term function are often underreported. Mitigation: Require post-application monitoring before broad extension.

**Table 4:** Minimum reporting standards for acidic soil diazotroph studies.

Field	Minimum Reporting Item	Purpose for Inference-Basis Classification
Soil pH	Measurement method, soil:solution ratio, depth, timing, and pre/post-inoculation values	Defines acid-soil context and separates pH effects from other constraints.
Exchangeable Al/Mn	Exchangeable or soluble Al and Mn, extraction method, and toxicity threshold if used	Tests whether metal toxicity constrains colonization or plant response.
Available P	Available P method and amendment history	Determines whether N fixation can translate into plant N assimilation and growth.
Organic matter	Organic matter or organic C, C/N ratio, and amendment inputs	Contextualizes carbon supply, buffering capacity, and microbial competition.
Host genotype	Crop species, cultivar/genotype, growth stage, and root phenotype endpoint	Allows host-demand and exudate effects to be interpreted.
Strain identity	Taxonomic identity, marker/genome evidence, accession or strain deposit, and functional traits	Prevents ambiguous attribution of diazotroph function.
Inoculum density	CFU, spores, carrier, formulation, application method, timing, and survival/shelf-life where relevant	Links dose and formulation to field persistence.
Colonization method	qPCR, tagged strain, reisolation, microscopy, *nifH* lineage tracking, or other validated method	Shows whether the organism reached and persisted in the target niche.
*nifH*/ARA/^15^N_2_ metric	nitrogenase reductase gene *nifH* abundance/expression, ARA units and incubation conditions, or ^15^N_2_ incorporation with controls	Distinguishes functional expression from trait presence.
Plant N/P uptake	Tissue N and P concentration/uptake, N-use efficiency, and isotope-derived N when possible	Connects microbial activity with plant nutrient acquisition.
Yield/root traits	Biomass, yield, root length/architecture, nodulation, stress markers, and sampling stage	Defines agronomic relevance beyond microbial endpoints.
Native microbiome shifts	16S/ITS/*nifH* profiles, diversity, network shifts, pathogen/beneficial guild changes, and target-strain persistence	Assesses microbiome compatibility and ecological risk.
Field duration	Pot/greenhouse/field status, site history, season, climate, replication, and multi-season follow-up	Separates short-term controlled efficacy from durable field performance.
Soil moisture	Water content, water potential, or irrigation status at sampling and during the study.	Interprets oxygen diffusion, solute transport, and microbial activity.
Sampling depth	Depth interval and whether bulk soil, rhizosphere soil, or root-associated material was sampled.	Defines the sampled niche and enables comparison across studies.
Root-exudate collection	Collection method, growth system, sampling time, and normalization approach where exudates are measured.	Allows recruitment and carbon-supply mechanisms to be interpreted.
Background nitrogen	Fertilizer form, rate, timing, and baseline soil N status.	Separates diazotroph responses from N-supply effects.
Weather and in-season conditions	Rainfall/irrigation, temperature, crop stage, and major stress events for field studies.	Interprets field heterogeneity and seasonal reproducibility.

## Data Availability

No new datasets were generated or analyzed for this narrative review. All information synthesized is available in the cited publications.
